# Job satisfaction among Community-Based Rehabilitation (CBR) workers in caring for disabled persons in the east coast region of Peninsular Malaysia

**DOI:** 10.1186/s12889-019-6520-z

**Published:** 2019-02-19

**Authors:** Haliza Hasan, Syed Mohamed Aljunid

**Affiliations:** 1 International Centre for Casemix and Clinical Coding, Faculty of Medicine, Universiti Kebangsaan Malaysia (National University of Malaysia), 56000 Cheras, Kuala Lumpur, Malaysia; 2 Department of Health Policy & Management, Faculty of Public Health, Kuwait University, Kuwait, Kuwait; 3 Department of Special Care Nursing, Kulliyyah of Nursing, International Islamic University Malaysia (IIUM), Jalan Hospital Campus, 25100 Kuantan, Pahang Malaysia

**Keywords:** Job satisfaction, Community-based rehabilitation, Community health workers, Disabled persons

## Abstract

**Background:**

Job satisfaction influences staff retention, motivation, and performance in providing services. A considerable amount of published studies has reported on the job satisfaction level of healthcare workers, but to date, very few studies focused on Community-Based Rehabilitation (CBR) workers. This study aimed to explore the job satisfaction level among Community-Based Rehabilitation (CBR) workers and associated factors related to their overall job satisfaction.

**Methods:**

A one-year survey was conducted in three states of the east coast region of Peninsular Malaysia involving 204 CBR workers selected through universal sampling method where all CBR staff who fulfilled the inclusion criteria were selected as participants. Self-completed questionnaires consisted of 20 association factors on six-point Likert scale responses were distributed. Total mean satisfaction level and mean associated factors were reported in this study.

**Results:**

The results showed that the majority of the participants were between 20 and 40 years old (72%), female (96%), Malay (99%) and had 1–5 years of working experience. The mean total satisfaction score was 79.8 ± SD = 7.85. The highest mean satisfaction level for the associated factor was 4.6 ± SD = 0.59 with about 95% of the participants were satisfied that *“CBR programme is a challenging work”,* while the lowest satisfaction level for associated factor was on *“salary of community-based rehabilitation staff is acceptable”,* with mean score of 2.3 ± SD = 0.97 with about 59% of the participants felt dissatisfied. The results of this study determined that the highest dissatisfied factors among CBR workers were on salary.

**Conclusion:**

These findings provided useful information for policymakers to evaluate this issue for a sustainable CBR programme in the future.

**Trial registration:**

This study has been registered for trial as ‘retrospective registered’ in the Australian New Zealand Clinical Trials Registry (ANZCTR) (registration no.: ACTRN 12618001101279) on 5th October 2018.

**Electronic supplementary material:**

The online version of this article (10.1186/s12889-019-6520-z) contains supplementary material, which is available to authorized users.

## Background

Research into job satisfaction has a long history. Over the past decade, most research in job satisfaction had emphasized it as the significant indicator used to measure the quality of services and to sustain the programme or service offered. Job satisfaction (JS) can be described as an attitude or personal perception towards their job experience, how they felt on their job; whether they are satisfied or dissatisfied with the job [1–3].

Satisfaction is one of the significant indicators to measure services provided in many sectors including healthcare providers, either primary, secondary or tertiary level. JS is always associated with quality of care and organization performance. Also found was that job satisfaction among healthcare workers showed a direct correlation between employees and patient satisfaction, prevent workers from turnover and warrant the sustainability of the services or programmes. Workers satisfaction may influence the retention rate with a particular organization and/or sustain the services or programmes offered by the organization [4].

Though, job satisfaction can be influenced by a variety of factors and should be discussed together as the factors were interrelated with each other [3]; this includes monetary and non-monetary factors [5]. The previous study underlined the main factor that influenced job satisfaction among workers was on competitive salary [6]. Salary was identified as one of the important factors associated with job satisfaction [7]. A study by Khamlub et al. (2013) also indicated that satisfaction level on salary factors showed many healthcare workers were dissatisfied with their salary and the lowest mean score was identified on this factor [2]. It indicated that monetary factor is a significant factor to be taken into consideration by the organization.

Other study suggested that although monetary factor contributed to influence job satisfaction, non-monetary factors also had significant influences [6]. Moreover, non-monetary factors comprised of several aspects including job environment, career development, management, relationship with colleagues, individual needs, heavy workload, working in shifts, high-risk work and lack of recognition [7, 8]. In 1999, Kim et al. found satisfied employees tend to be more productive and creative [8]. In another major study, Kinzl et al. (2005) found that job satisfaction had a greater relationship with stress among workers [9]. Although job satisfaction had been discussed in many working fields, to date, there are few studies that have explored job satisfaction among community-based rehabilitation (CBR) workers.

Community-Based Rehabilitation (CBR) programme is one of the World Health Organization’s initiatives through combined efforts of various relevant parties including people with disabilities, their families, communities, government and non-government health organizations, education, vocational and social institutions as well as other services to assist disabled people in community settings [10]. Worldwide, more than 90 countries have implemented CBR programmes to cater for people with disabilities in a large population [11] including Malaysia [12]. Malaysia had implemented the first CBR programme since 1984 in Batu Rakit, Terengganu in collaboration with the Ministry of Health Malaysia. CBR programme focuses on long-term care and acts as a training centre that prepares disabled persons to enrol in special schools, employment and consequently live in the community with the highest possible capability [13, 14]. The CBR centre operated from 8.00 a.m. until 2 p.m., from Monday to Friday. The activities comprised of Circle time, exercise, group activity, individual activity, music, basic religious (fardhu ain), visited staff from the medical team, dental team, physiotherapist, occupational therapist, dietician and teacher for disabled children [14].

The prevalence of persons with a disability had increased since the last two decades from approximately 10% in the 1970s to 15% in 2011, and approximately 2–4% of the world population experienced some form of disabilities [15, 16]. The increasing number of disabled children worldwide also contribute to the statistic of persons with disability, albeit a relatively small proportion of the statistic. Malaysia as a developing country is also concerned with this increasing number of disabled persons. In the year 2012, approximately 445,006 people with various forms of disabilities have registered with The Department of Social Welfare Malaysia (DSWM) which represents 1.5% of the country’s population. Also reported, that 0.1% out of 1.5% of the country’s population was contributed from 29,289 children [17]. This proportion of the population needs long-term care and it is highlighted that job satisfaction among CBR workers is significant to be evaluated due to the scarcity to find people who are willing to work with this special population.

Therefore, this study was conducted to explore JS level among CBR workers in giving care for disabled persons in Malaysia. An established Job Satisfaction Survey (JSS) scale was chosen as the instrument to measure the JS level among the CBR workers.

## Methods

### Study design and study area

A cross-sectional study design was conducted from April 2015 to April 2016 in the three states of the east coast region of Peninsular Malaysia; Pahang, Terengganu and Kelantan. Community-based rehabilitation centres are rehabilitation centres that cater for disabled persons from ages 2 to 45 in a community setting. The Community-based rehabilitation centres were operated five days per week from 8.00 a.m. until 2.00 p.m. There were several activities provided by the centres which were circle time, music therapy, art therapy, individual therapy, group therapy, gardening, cooking therapy, monthly routine medical check-up, dental routine visit, physiotherapy and occupational therapy.

### Study population and sampling

The participants were recruited using the universal sampling method. This study is part of a whole economic evaluation study conducted by the researchers. All CBR workers who fulfilled the inclusion criteria during data collection period were selected as study samples. The inclusions such as local citizen, working experience for at least 6 months and understand Malay and English Language were underlined for sampling determinations. The minimum period of 6-month working experience was set up as the cut-off point to determine the inclusion criteria based on the local practice in Malaysia. The 222 CBR workers who fulfilled the inclusion criteria from selected study locations were invited to participate. From a total of 222 satisfaction questionnaires distributed, 204 questionnaires were returned completely (response rate was 92%).

### Instrument

The questionnaire comprised of two parts which were demography and job satisfaction survey. An additional questionnaire file shows the contents in more detail [see Additional file 1]. Demography information including gender, ethnic, age, educational level, work experience and position was in part I, while part II was the job satisfaction survey (JSS) which consisted of 20 influencing factors such as CBR schedule and location, service provision, salary, information dissemination, use of skills and abilities, respect for trainees and individual attention, punctuality, meaningful work, challenging job, stress, opportunities, job pride, understanding on trainees’ condition, respectful of ethnicity, impact to other people, encouragements and overall satisfaction. The JSS used the six-point Likert scale representing various degrees of satisfaction: 0 = no opinion, 1 = very dissatisfied, 2 = dissatisfied, 3 = moderate, 4 = satisfied, and 5 = very satisfied. Questions in this part were developed from a published study by Goldstein and colleagues in the year 2000 and were purposely created for this study. The tool was proven to be highly reliable with reported coefficient of Cronbach’s alpha of 0.99 [18]. Since the questionnaire has not been used in Malaysia before, the researchers conducted a pilot study to test for its reliability in the local settings. The coefficient for reliability analysis obtained Cronbach’s alpha of 0.871 for the instrument and was clearly within a desired range. Findings from the pilot study showed that the questionnaire is reliable to be used in local settings.

### Statistical analysis

All data obtained were analysed using Statistical Package for the Social Science (SPSS) Window version 20. Descriptive statistic was used and results were presented in mean score for each factor. Pearson Correlation was used to test for statistically significant relationship between satisfaction score and age of the participants, *One-way ANOVA* test was used to identify the relationship between satisfaction score with education level and working experience and independent t-test analysed the relationship between satisfaction scores and position of the participants. The overall JSS score is classified as dissatisfied, moderate, satisfied and no opinion with total scores of 20–40, 41–60, 61–100 and 0 respectively [1]. *P*-value < 0.05 was considered as statistically significant.

### Pilot study

Fifteen CBR staff were recruited as participants in the pilot study. Participants demonstrated their consent by signing the consent form before the questionnaires were distributed to all agreed participants. Briefing session was conducted that focused on the consistency of the questionnaires. This is to ensure that the questionnaire was appropriate, well defined, clearly understood and presented in a consistent manner before all participants answered the questions. The questionnaire assessed 20 satisfaction factors which were divided into five dimensions of satisfaction which were “access” (2 factors), “administrative technical management” (2 factors), “clinical technical management” (2 factors), “interpersonal management” (11 factors) and “continuity of care” (3 factors). The combination from all dimensions constitutes the overall job satisfaction of CBR workers. The Likert scale was modified to suit this study purpose ranging from 0 point (no opinion) to 5 points (very satisfied). The overall level of job satisfaction score was derived from the sum of responses for all 20 items. The overall levels of job satisfaction among CBR workers were classified as being dissatisfied if the sum of the score was 20–40, while 41–60 was moderate and 61–100 was satisfied [1]. The questionnaire has been translated and validated in the Malay language by a language expert from Centre for Languages and Pre-University Academic Development (CELPAD), International Islamic University Malaysia. The questionnaire was produced in both the Malay and English languages.

Issues that were observed among the participants in the pilot study included the ability to understand the instructions, understanding of questionnaire items, the terms, the sequence of questions and the flow of statements, the format including font and layout, as well as length of time to complete the questionnaire. The feasibility of the study instrument was observed for the time taken by the researchers to explain the research project and to obtain consent from agreed sample. The time taken to explain the research study was approximately 2–3 min, while time taken for answering the questionnaires took approximately 8–10 min. Most of the questions were considered as acceptable and understandable and participants were allowed to ask the researchers in case of any doubt. All questionnaires returned were checked by the researchers. The coefficient for reliability analysis obtained Cronbach’s alpha of 0.871 for the instrument and was clearly within a desired range. Findings from the pilot study show that the questionnaire is reliable to be used in local settings.

## Results

### Socio-demographic status of participants

A total of 204 participants were recruited in this study. The number of participants from Pahang, Terengganu and Kelantan were 60 participants (29%), 82 participants (40%) and 62 participants (30%), respectively, with a mean score of 2.0 ± 0.77. The majority of the participants were female which was 95% (195 participants) and 4% were male (9 participants). Only one (1%) respondent was from Chinese ethnic, while 203 participants were Malay (99%). Age groups of the participants were categorised by 20–30 years old (35%), 31–40 years old (35%), 41–50 years old (23%) and 51–60 years old (5%). Most of the CBR staff completed secondary school (75%), 44 participants (22%) had tertiary education level and 7 participants (3%) completed primary school only.

Most of the participants had working experience between 1 and 5 years (39%), while working experience between 16 and 20 years was the lowest percentage (5%). About 33 participants were supervisors (16%) and 171 participants were trainers (84%). The demographic characteristics of the CBR staff are shown in Table 1.

**Table 1:** Demographic characteristics of the CBR workers (*N* = 204)

Characteristics	N	*N* (%)
States		
Pahang	60	(29)
Terengganu	82	(40)
Kelantan	62	(30)
Age groups		
20–30 years old	73	(36)
31–40 years old	73	(36)
41–50 years old	47	(23)
51–60 years old	11	(5)
Ethnic		
Malay	203	(99)
Chinese	1	(1)
Gender		
Female	195	(96)
Male	9	(4)
Education levels		
Primary school	7	(3)
Secondary school	153	(75)
College/university	44	(22)
Working experience		
6 months - < 1 year	11	(5)
1–5 years	79	(39)
6–10 years	58	(29)
11–15 years	34	(17)
16–20 years	10	(5)
> 20 years	12	(6)
Position		
Supervisor	33	(16)
Trainer	171	(84)

### Factors associated with job satisfaction

Table 2 shows the CBR workers’ rates of job satisfaction among various factors. Analysis of the job satisfaction by associated factors among CBR workers had shown that the highest mean satisfaction level was 4.6 ± 0.59 with 194 participants (95%) felt satisfied that *“CBR programme is a challenging work”* while 9 participants (4%) were dissatisfied and only 1 respondent (1%) felt moderate satisfaction with this factor. The lowest mean satisfaction for influencing factor was 2.3 ± 0.97 on the factor of *“salary of CBR staff is acceptable”* where 28 participants (14%) were satisfied, 53 participants (26%) had moderate satisfaction, 121 participants (59%) were dissatisfied with the salary obtained and 2 participants (1%) had no opinion for this factor. Five influencing factors were found to have mean scores between 3.0 to 4.0. The mean for the influencing factor of *“providing rehabilitation services as promised”* was 3.85 ± 0.78; 155 participants (76%) were satisfied with this factor, and guaranteed providing good services, 42 participants (21%) expressed moderate satisfaction, 5 participants (3%) were dissatisfied and 2 participants (1%) had no opinion if the services provided met their promise. Mean score for factor *“my job makes good use of my skills and abilities”* was 3.9 ± 0.65; 159 participants (78%) were satisfied, 45 participants (22%) had moderate satisfaction and no participants expressed their dissatisfaction. Factor of *“giving trainees individual attention”* had a mean of 3.7 ± 1.45 with 163 participants (80%) were satisfied, 14 participants (7%) had moderate satisfaction, 5 participants (3%) were dissatisfied and 22 participants (11%) had no opinion on this factor. Mean score for factor *“there are opportunities for me while working in CBR programme”* was 3.4 ± 1.46 (136 participants were satisfied, 35 participants had moderate satisfaction, 10 participants were dissatisfied, and 23 participants had no opinion).

**Table 2:** Association factor of job satisfaction (N = 204)

Variables	Mean ± SD	Dissatisfied Score 20–40 N (%)	Moderate Score 41–60 N (%)	Satisfied Score 61–100 N (%)	No opinion Score 0 N (%)
Q1. The CBR Centre scheduled at convenient times.	4.3 ± 0.57	–	12 (6)	192 (94)	–
Q2. The location of the CBR centre was convenient for me.	4.3 ± 0.58	1 (1)	11 (5)	192 (94)	–
Q3. Provided rehabilitation services as promised.	3.9 ± 0.78	5 (3)	42 (21)	155 (76)	2 (1)
Q4. The salary for CBR workers was acceptable.	2.3 ± 0.97	121 (60)	53 (26)	28 (13)	2 (1)
Q5. Keeping trainee and/or family informed about CBR services.	4.2 ± 0.57	–	19 (9)	185 (91)	–
Q6. My job makes good use of my skills and abilities.	4.0 ± 0.65	–	45 (22)	159 (78)	–
Q7. Always respect trainee during CBR programme	4.3 ± 0.68	1 (1)	15 (7)	187 (92)	1 (1)
Q8. Giving trainees individual attention.	3.7 ± 1.45	5 (3)	14 (7)	163 (80)	22 (11)
Q9. I am conduct the CBR program on time.	4.1 ± 0.69	5 (3)	19 (9)	179 (88)	1 (1)
Q10. CBR programme is meaningful work to me.	4.4 ± 0.5	–	9 (4)	195 (96)	–
Q11. CBR programme is a challenging job.	4.6 ± 0.59	1 (1)	9 (4)	194 (95)	–
Q12. I often felt stressed at work.	2.8 ± 0.96	63 (31)	108 (53)	33 (16)	–
Q13. There were some opportunities to me for working in CBR programme.	3.5 ± 1.46	10 (5)	35 (17)	136 (67)	23 (11)
Q14. I am proud being one of the CBR trainers.	4.4 ± 0.63	–	6 (3)	197 (97)	1 (1)
Q15. I understood my trainee’s problem and condition.	4.2 ± 0.59	2 (1)	12 (6)	190 (93)	–
Q16. I understand why it is so important for me to recognize and respect the value of differences in race, gender, age, etc.	4.3 ± 0.62	–	7 (3)	196 (96)	1 (1)
Q17. My job makes a difference in the lives of others.	4.0 ± 0.89	11 (5)	26 (12)	164 (80.9)	2 (1.0)
Q18. I satisfied with my employment benefits	4.1 ± 0.70	4 (2)	23 (11)	176 (86.3)	1 (1)
Q19. I feel encouraged to come up with new and better ways of doing CBR programme.	4.3 ± 0.68	1 (0.5)	16 (7.8)	186 (91.2)	1 (0.5)
Q20. Overall, I was satisfied with my job as a CBR trainer	4.3 ± 0.61	–	17 (8.3)	187 (91.7)	–

The mean overall job satisfaction of CBR workers in CBR programme was 79.8 ± 7.85 with 204 participants (100%) were satisfied with their job as CBR workers (Table 3).

**Table 3:** Total job satisfaction score of CBR workers (N = 204)

Variable	Mean ± SD	*N* (%)
Total satisfaction score	79.8 ± 7.85	
Dissatisfied (score 20–40)		–
Moderate (score 41–60)		–
Satisfied (score 61–100)		204 (100)

### Association job satisfaction between satisfaction score and demographic characteristics

Job satisfaction may be influenced by demographic characteristics of the participants. Table 4 shows the association between satisfaction score and demographic characteristics of the participants. *One-way ANOVA* test was used to identify the relationship between satisfaction score and educational level, working experience, gender as well as satisfaction level between states with no significant relationship found between the tested variables; educational level (*F* = 0.006, *p* = 0.99), working experience (*F* = 0.09, *p* = 0.23), gender (*t* = − 0.57, *p* = 0.57) and between states (*F* = 2.38, *p* = 0.095). However, *independent t-test* showed that satisfaction score between position was significantly higher among supervisors (83.15 ± 7.17) than trainers (79.18 ± 7.83) with *t* = 2.71, *p* = 0.007.

**Table 4:** Association between demographic characteristics and satisfaction score of CBR workers

Variables	Satisfaction score Mean (SD)	Test- value	*p*-value
Working experience		0.09^b^	0.203
Educational level		0.006^b^	0.994
Primary school	82.7 (4.96)		
Secondary school	79.4 (8.02)		
College/ University	80.8 (7.55)		
State		2.38^b^	0.095
Pahang	81.1 (7.16)		
Terengganu	80.2 (8.06)		
Kelantan	78.1 (8.03)		
Position		2.71^c^	0.007^a^
Supervisor	83.2 (7.17)		
Workers	79.2 (7.83)		
Gender		-0.566^c^	0.572
Male	80.1 (5.67)		
Female	78.8 (6.59)		

^a^Significant at 0.05

^a^Pearson correlation, ^b^ one-way ANOVA, ^c^ independent t-test

## Discussion

### Factors associated with job satisfaction

Job satisfaction in CBR workers is important for a sustainable long-term care for disabled persons but still lacked attention on this issue. Hence, this study was conducted to examine the level of satisfaction among CBR workers.

In this study, 20 associated satisfaction factors were highlighted and divided into 5 domains which were “*access*”, “*administrative technical management*”, “*clinical management*”, “*interpersonal management*” and “*continuity of care*” that may influence satisfaction level of CBR workers. Results showed that the highest level of satisfaction among CBR workers was on the factor of “*CBR programme is a challenging job*”. Almost all CBR workers were satisfied with their job and expressed that working in CBR programme requires high responsibility because they are dealing with persons with different abilities. Similar to previous studies, challenges in carrying out the responsibility and multitasking works was identified as one of the influence factor of job satisfaction [19, 20]. This shows that responsibility is a positive factor of job satisfaction among CBR workers. Multitasking or mismatching of work may cause dissatisfaction among them.

‘*Salary*’ or ‘*payment*’ or ‘*pay*’ is the frequent factor assessed for job satisfaction. In this study, we found that ‘*salary*’ or ‘*pay*’ is the lowest satisfaction factor. This result is similar to the study done by Ding et al. [20]. Job satisfaction factor of ‘*your rate of pay’* was also highlighted in the study of job satisfaction and motivation among Malaysian primary healthcare professionals by Chew et al. [21]. Analysis of literature from the previous studies found salary as one of the main factors that influence satisfaction among healthcare and social workers [19–21]. In comparison, a study in Vietnam found that financial and non-financial factors influence job motivation and satisfaction among rural health workers [5]. Those workers, for which remuneration is the most important factor, are more likely to find other jobs that pay for higher salary and this would contribute to staff turnover [19]. Hence, the salary factor is a vital point in identifying satisfaction related issues because it involves not only healthcare services but also in a production organization. Workers wage was also highlighted to have a positive correlation with workers’ wellbeing in a production organization setting and salary as an incentive influences job satisfaction in every level of the profession [22]. The low salary in CBR centre discourages the community to participate as CBR workers. This study shows that although the level of satisfaction on salary incentive was the lowest, the mean satisfaction level was higher in the factor of “providing rehabilitation services as promised”. Results of the study revealed that most of the CBR workers did not feel that the work was stressful and still provided the best services as promised to disabled persons in the CBR programme (Table 1). This supports the study by Peters et al. [23] that more workers expressed that satisfaction and motivation factors were more important than income. The findings contradicted the study by Ding et al. that suggested low salary as one the most frequent influence factor to job stressor in a workplace [21].

Other significant implications of factors observed were *‘my job makes good use of my skills and abilities’*, *‘giving trainees individual attention’*, ‘I often felt stressed at work’ and *‘there are opportunities for me while working in CBR programme’* which seemed to be fair to CBR workers. In contrast, the study by Ge et al. (2011) indicated that stress was the highest influence factor to job satisfaction among community health workers [3]. Further research on these factors needs added attention to examine precisely the cause of job satisfaction and to obtain accurate responses from CBR workers.

Other satisfaction factors observed seemed to be satisfactory to most of the CBR workers (mean > 4.0). These analysed items were comparable to study by Peters et al. (2010) that percentages of satisfaction for each factor were found to be more than 80% for each satisfaction factors(23) [23]. Mean overall satisfaction score of CBR workers was 79.82 ± 7.85 with surprisingly all of the 204 respondents were satisfied with their job.

### Association job satisfaction between satisfaction score and demographic characteristics

In this study, all CBR workers were assigned randomly to carry out their job assignment to reduce bias for the assessment of estimating the impact of job task with the satisfaction level among them. Inappropriate identification of workers’ satisfaction factors may increase bias in determining the impact of job on the satisfaction and wellbeing of the workers. An advanced method was implemented in a study by Böckerman and colleagues (2012) through a combination of survey and formal documentation which was identified as an effective approach in estimating workers’ wellbeing [22]. However, the methods were not applied in the present study due to documentation limitation in CBR centres.

In the present study, no significant association between participant’s demographic characteristics such as the level of education, duration of working experience, state of residence and gender with the level of job satisfaction were found. This finding was also similar to the study by Boston (2008) that years of working experience and gender did not significantly influence the job satisfaction of workers. In contrast, many other studies found that the duration of working experience influenced staff satisfaction level and motivation [19, 24].

Work history including absenteeism can also influence workers’ satisfaction and happiness with their job. This factor was highlighted in the study by BÖckerman and colleague (2012) which found that a history of sickness had affected workers wellbeing and satisfaction [22]. However, there is a limited discussion on absenteeism issue hindered by scarce references that are difficult to identify in literature or an inadequate strategy to identify the references. In the present study, history of absence was not used to identify their impact on satisfaction level of CBR workers as there was a limitation in the documentation process and had limited references. Other demographic data that were mostly discussed in previous literature were used for the identification of job impact to satisfaction level.

The present study found that there was a significant association between the job satisfaction levels and the CBR workers’ position. The satisfaction scores were significantly different between supervisors and trainers. Satisfaction level of supervisors was higher compared to trainers (*p* = 0.007) (Table 4). This relationship could be possibly related in the differences of salary earned between supervisors and trainers. The difference of salary between these two groups of CBR workers may influence satisfaction among them. Similarly, previous studies suggested that those holding managerial positions were more likely to have higher satisfaction and motivation compared to other co-workers [22, 23, 25]. The study by Boston (2008) stated that a position’s workload is also significantly associated with the level of satisfaction among social health workers, where the ordinary workers illustrated low satisfaction level compared to the higher level of workers [26]. The findings from the literature and the present study showed that a worker’s position had a positive impact on job satisfaction among social health workers. The responsibility of the job may influence their satisfaction level.

### Limitations

Limitations in this study were identified from the questionnaire used and study areas. More influences factors related to the job satisfaction of CBR workers should be examined for genuine responses from the participants, including a history of absence. The study location should be expended to other areas of Malaysia in order to obtain a broader view of workers towards their job, although there was no issue on the participant’s bias. Almost all participants were female but many of the previous study done had a majority of female CBR workers. It is a norm in Malaysia that the majority of healthcare workers are dominated by females.

## Conclusions

In conclusion, surprisingly all of CBR workers were satisfied with their job to serve for a disabled person. Though, we found that low satisfaction level on salary and challenging work among CBR workers did not influence the services provided. Although the mean score was low on the factor of ‘the salary of CBR workers was acceptable’, but the mean score was high on the factor of ‘providing rehabilitation services as promised’ which indicated that the participants felt they provided rehabilitation services of good quality despite being dissatisfied with their salary. Thus, this study provides empirical evidence that salary and challenging work were negatively associated with job satisfaction.

Nevertheless, findings in this study did not explore all outcome that may have been demonstrated by the CBR workers towards their job satisfaction. Limited published reviews related to our study made the referencing difficult. Practical policies such as workplace wellbeing, workplace absence and workplace performance should be examined to identify the effect of job satisfaction among CBR workers in our future research.

## Recommendation

The study findings could help policymakers to improve and sustain CBR programme by giving attention to the factors that influence job satisfaction and motivation among CBR workers. Monetary and non-monetary incentives such as the revision of salary scheme, career advancement opportunities, and provision of specific training in caring for disabled persons should be given attention to address job dissatisfaction among CBR workers. The findings from this study that was conducted among CBR workers from the east coast region of Peninsular Malaysia could be generalised to all CBR workers in the country and provide evidence to other countries that have implemented CBR programmes.

## Additional file

Additional file 1: Questionnaire on job satisfaction. This questionnaire consisted of 20 questions and divided into five sections which were ‘Access’, ‘Administrative Technical Management’, ‘Clinical Management’, ‘Interpersonal Management’ and ‘Continuity of Care’ that related to satisfaction for CBR workers. (PDF 769 kb)

## Abbreviations

ANOVA: Analysis of Variance
CBR: Community-Based Rehabilitation
CELPAD: Centre for Languages and Pre-University Academic Development
DSWM: Department of Social Welfare Malaysia
JS: Job Satisfaction
JSS: Job Satisfaction Survey
SD: Standard Deviation
