# Supplementary material for: Job satisfaction among Community-Based Rehabilitation (CBR) workers in caring for disabled persons in the east coast region of Peninsular Malaysia
Source: BMC Public Health. 2019 Feb 19;19:208. doi: 10.1186/s12889-019-6520-z (PMC6381719; doi:10.1186/s12889-019-6520-z)
Supplement: Supplementary file 1 — Questionnaire on job satisfaction. This questionnaire consisted of 20 questions and divided into five sections which were ‘Access’, ‘Administrative Technical Management’, ‘Clinical Management’, ‘Interpersonal Management’ and ‘Continuity of Care’ that related to satisfaction for CBR workers. (PDF 769 kb) [file 12889_2019_6520_MOESM1_ESM.pdf]

Participant ID:……………..

CBR ID.:…………….

# FORM 3B

**Tajuk Kajian: Kajian Keberkesanan Kos Program Pemulihan dalam Komuniti (PDK) untuk Kanak-kanak Kurang Upaya di Malaysia: Perbandingan Antara Penjagaan di Pusat Pemulihan dengan Penjagaan di Rumah**

*Research Title: Cost-effectiveness of Community-Based Rehabilitation (CBR) Programme for Disabled Children in Malaysia: A Comparison Between Centre-based and Home-based Care*

**Tinjauan Kepuasan Kakitangan**

*Staff Satisfaction Survey*

*Sekiranya ada sebarang soalan, sila hubungi:*
*If you have any concerns please contact:*

1. *Haliza Hasan – 012 987 1404 (hhaliza80@gmail.com)*
2. *Prof. Dato'. Dr. Syed Mohamed Aljunid Syed Junid – 012 638 3521(saljunid@gmail.com)*

**Kami berminat untuk mengetahui bagaimana anda berpuas hati dengan Program Pemulihan Dalam Komuniti (PDK) sebagai pelatih dan kakitangan Jabatan Kebajikan Masyarakat, Malaysia**.

*We are interested in knowing how you are satisfied with Community-Based Rehabilitation (CBR) programe as a trainer and staff of Department of Social Welfare, Malaysia.*

**Sila jawab soalan-soalan berdasarkan pengalaman anda dengan program PDK dengan menanda (√) di kotak yang disediakan**

*Please answers the questions based on your experience with CBR programme by marks (√) in the boxes provided*

1. **Bagaimana anda memohon kerja anda di jabatan ini?**
   *How did you apply your job in this department?*

☐ Kawan *Friend* ☐ Surat khabar *News paper*

☐ Laman web *Website* ☐ Lain-lain, sila nyatakan…………………..
*Others, please indicate……………..*

2. **Adakah ini pengalaman pertama anda bekerja dengan terapi di PDK?**
   *Was this your first experience working with CBR therapy?*

☐ Ya *Yes* ☐ Tidak *No*

**3. Sila tandakan jenis kelainan upaya yang ditugaskan kepada anda untuk anda melakukan terapi di PDK (Anda boleh tanda lebih daripada satu).**
*Please tick which of the disabilities that you are assigned for therapy in CBR programme (You may tick more than one)*

☐ Kurang upaya penglihatan *Visually impaired* ☐ Kurang upaya pendengaran *Hearing impaired*

☐ Kurang upaya fizikal *Physically disabled* ☐ Kurang upaya pembelajaran *Learning disabilities*

☐ Kurang upaya pertuturan *Speech disabilities* ☐ Mental *Mental*

☐ Kurang upaya pelbagai *Multiple disabilities*

1. **Sila baca kenyataan yang diberikan**
   Please read the statement given

2. **Gunakan skala yang diberikan bagi menyatakan tahap kepuasan anda pada setiap pernyataan berikut**
   Use the scale given to rate your degree of satisfaction with each of the following statements

3. **Sila tandakan (√) dalam petak berkenaan**
   *Kindly please (√) appropriate respond.*

## Skala Tahap Puas Hati
*Degree of Satisfaction*

| 1 | 2 | 3 | 4 | 5 | 0 |
|---|---|---|---|---|---|
| **Sangat tidak setuju** *Strongly dissatisfied* | **Tidak setuju** *Dissatisfied* | **Neutral** *Neutral* | **Setuju** *Satisfied* | **Sangat setuju** *Strongly satisfied* | **Tidak mempunyai pendapat** *No opinion* |

| **Bil.** No. | **Item** *Item* | **Skala** *Scale* | | | | | |
|---|---|---|---|---|---|---|---|
| | | **1** | **2** | **3** | **4** | **5** | **0** |
| **A.** | **Akses** *Access* | | | | | | |
| **1.** | **Pusat PDK dijadualkan pada waktu yang sesuai.** *The CBR Centre scheduled at convenient times.* | | | | | | |
| **2.** | **Lokasi pusat PDK adalah mudah untuk saya.** *The location of the CBR centre is convenient for me.* | | | | | | |
| **B.** | **Pengurusan Pentadbiran Teknikal** *Administrative Technical management* | | | | | | |
| **3.** | **Menyediakan perkhidmatan pemulihan seperti yang dijanjikan.** *Providing rehabilitation services as promised.* | | | | | | |
| **4.** | **Gaji untuk pekerja PDK adalah berpatutan**. *The salary for CBR staff was acceptable.* | | | | | | |
| **C.** | **Pengurusan Klinikal** *Clinical Management* | | | | | | |
| **5.** | **Sentiasa memaklumkan tentang perkhidmatan PDK kepada pelatih dan keluarga.** *Keeping trainee or/ and family informed about CBR services.* | | | | | | |
| **6.** | **Pekerjaan saya banyak menggunakan kemahiran dan kebolehan saya**. *My job makes good use of my skills and abilities.* | | | | | | |
| **D.** | **Pengurusan Interpersonal** *Interpersonal Management* | | | | | | |
| **7.** | **Sentiasa menghormati pelatih semasa program PDK dijalankan**. *Always respect trainee during CBR programme.* | | | | | | |

| | | | | | | | |
|---|---|---|---|---|---|---|---|
| **8.** | **Memberi perhatian individu kepada pelatih.**<br>*Giving trainees individual attention.* | | | | | | |
| **9.** | **Saya menepati masa dalam mejalankan program PDK.**<br>*I am punctually conducting the CBR programme.* | | | | | | |
| **10.** | **Program PDK merupakan pekerjaan yang bermakna kepada saya.**<br>*CBR programme is meaningful work to me.* | | | | | | |
| **11.** | **Program PDK adalah tugas mencabar.**<br>*CBR programme is a challenging job.* | | | | | | |
| **12.** | **Saya sering berasa stres di tempat kerja.**<br>*I often feel stressful at work.* | | | | | | |
| **13.** | **Terdapat banyak peluang kepada saya semasa bekerja di dalam program PDK**.<br>*There are an opportunities for me while working in CBR programme.* | | | | | | |
| **14.** | **Saya bangga menjadi salah seorang petugas PDK.**<br>*I am proud of being one of the CBR trainers.* | | | | | | |
| **15.** | **Saya memahami masalah dan keadaan.pelatih saya.**<br>*I understood my trainee's problem and condition*. | | | | | | |
| **16.** | **Saya memahami bahawa sangat penting bagi saya untuk mengenali dan menghormati nilai perbezaan kaum, jantina, umur, dan lain-lain.**<br>*I understand why it is so important for me to recognise and respect the value of differences in race, gender, age, etc.* | | | | | | |
| **17.** | **Pekerjaan saya memberi perbezaan dalam kehidupan orang lain**.<br>*My job makes a difference in the lives of others.* | | | | | | |
| **E.** | **Penjagaan Berterusan**<br>*Continuity of Care* | | | | | | |
| **18.** | **Saya berpuas hati dengan manfaat pekerjaan yang diperolehi .**<br>*I am satisfied with my employment benefits.* | | | | | | |
| **19.** | **Saya berasa digalakkan untuk menghasilkan cara baru yang lebih baik untuk menjalankan program PDK.**<br>*I feel encouraged to come up with new and better ways of doing CBR programme.* | | | | | | |
| **20.** | **Secara keseluruhan, saya berpuas hati dengan tugas saya sebagai petugas PDK.**<br>*Overall, I am satisfied with my job as a CBR trainer.* | | | | | | |

## - TERIMA KASIH -

***- THANK YOU -***
